# Functional role and tobacco smoking effects on methylation of *CYP1A1* gene in prostate cancer

**DOI:** 10.18632/oncotarget.9470

**Published:** 2016-05-19

**Authors:** Yozo Mitsui, Inik Chang, Taku Kato, Yutaka Hashimoto, Soichiro Yamamura, Shinichiro Fukuhara, Darryn K. Wong, Marisa Shiina, Mitsuho Imai-Sumida, Shahana Majid, Sharanjot Saini, Hiroaki Shiina, Koichi Nakajima, Guoren Deng, Rajvir Dahiya, Yuichiro Tanaka

**Affiliations:** ^1^ Department of Surgery/Urology, Veterans Affairs Health Care System, San Francisco, California 94121, USA; ^2^ Department of Urology, University of California, San Francisco, California 94121, USA; ^3^ Department of Urology, Shimane University Faculty of Medicine, Izumo, 693-8501, Japan; ^4^ Department of Oral Biology, Yonsei University College of Density, Seoul, 120-752, South Korea; ^5^ Department of Urology, Osaka University Graduate School of Medicine, Suita, 565-0871, Japan; ^6^ Department of Urology, Toho University Faculty of Medicine, Tokyo, 143-8540, Japan

**Keywords:** cytochrome P450 1A1, methylation, prostate cancer, tobacco smoking

## Abstract

*Cytochrome P450 (CYP)* 1A1 is a phase I enzyme that can activate various compounds into reactive forms and thus, may contribute to carcinogenesis. In this study, we investigated the expression, methylation status, and functional role of *CYP1A1* on prostate cancer cells. Increased expression of *CYP1A1* was observed in all cancer lines (PC-3, LNCaP, and DU145) compared to BPH-1 (*P* < 0.05); and was enhanced further by 5-aza-2′-deoxycytidine treatment (*P* < 0.01). Methylation-specific PCR (MSP) and sequencing of bisulfite-modified DNA of the xenobiotic response element (XRE) enhancer site XRE-1383 indicated promoter methylation as a regulator of *CYP1A1* expression. In tissue, microarrays showed higher immunostaining of *CYP1A1* in prostate cancer than normal and benign prostatic hyperplasia (BPH; *P* < 0.001), and methylation analyses in clinical specimens revealed significantly lower methylation levels in cancer compared to BPH at all enhancer sites analyzed (XRE-1383, XRE-983, XRE-895; *P* < 0.01). Interestingly, smoking affected the XRE-1383 site where the methylation level was much lower in cancer tissues from smokers than non-smokers (*P* < 0.05). *CYP1A1* levels are thus increased in prostate cancer and to determine the functional effect of *CYP1A1* on cells, we depleted the gene in LNCaP and DU145 by siRNA. We observe that CYP1A1 knockdown decreased cell proliferation (*P* < 0.05) and increased apoptosis (*P* < 0.01) in both cell lines. We analyzed genes affected by CYP1A1 silencing and found that apoptosis-related *BCL2* was significantly down-regulated. This study supports an oncogenic role for *CYP1A1* in prostate cancer via promoter hypomethylation that is influenced by tobacco smoking, indicating *CYP1A1* to be a promising target for prostate cancer treatment.

## INTRODUCTION

Prostate cancer is the most frequently diagnosed malignancy and the second leading cause of cancer death among men in the United States [1]. It is estimated that in the year 2016, there will be 180,890 new cases and 26,120 deaths due to prostate cancer [1]. This cancer is a disease of aging as 1 in 325 persons will develop invasive prostate cancer prior to the age of 50, but drastically rises to 1 in 48 for those aged 50 to 59, 1 in 17 aged 60 to 69, and 1 in 10 aged 70 years and older [1]. Despite these high rates, the genetic basis of this disease is not well understood.

Recent meta-analysis showed a close association between several *cytochrome P450 (CYP) 1A1* polymorphisms and prostate cancer risk [2, 3], suggesting that *CYP1A1* may contribute to prostate cancer tumorigenesis. The carcinogenic potential of *CYP1A1* is thought to be associated with metabolic activation of procarcinogens such as polycyclic aromatic hydrocarbons (PAHs), which can form PAH-DNA adducts in several types of malignancies [4, 5]. Although PAHs produce significant levels of PAH-DNA adducts in prostate cancer cells, its correlation with prostate cancer risk is controversial [6, 7]. Interestingly, recent studies have shown that *CYP1A1* promotes breast cancer progression even in the absence of xenobiotics [8] and suggests the possibility that this gene may be involved in other carcinogenic mechanisms.

*CYP1A1* is located in chromosome 15q24.1 region, consists of 7 exons and is roughly 6 kilobases in length. The protein localizes mainly to the endoplasmic reticulum and is composed of 512 amino acids with a size of 58 kDa. Under normal physiologic conditions, *CYP1A1* expression is induced by PAHs via activation of the aryl hydrocarbon receptor (AhR). The AhR complex then translocates to the nucleus and binds to its partner protein, aryl hydrocarbon receptor nuclear translocator (ARNT). The AhR/ARNT heterodimer binds to specific DNA recognition sites termed xenobiotic responsive elements (XREs) located upstream of the transcription start site and initiates *CYP1A1* transcription [3, 9]. Thus *CYP1A1* expression is regulated by direct interaction between the AhR/ARNT heterodimer and XREs.

Studies have shown that epigenetic changes can regulate the expression of several tumor-specific genes [10, 11]. We have demonstrated that DNA hypermethylation of CpG islands involving the promoter of tumor suppressor genes can lead to functional loss of these genes in several types of malignancies, including prostate cancer [12–14]. Also, DNA hypomethylation of oncogenic genes is thought to be associated with prostate cancer development and progression [15]. Previous studies have shown that the expression level of *CYP1A1* is frequently up-regulated in several human tissues due to hypomethylation of XRE sites which may promote binding of the AhR/ARNT heterodimer [16–19].

One putative mechanism affecting XRE methylation status of *CYP1A1* is tobacco smoking as demonstrated in human lung [17, 18]. It is widely accepted that tobacco smoking can cause lung cancer, and smoking-induced *CYP1A1* gene alterations may contribute to the initiation of lung carcinogenesis [17, 18]. Importantly, recent studies have shown a close association of smoking with the risk of prostate cancer [20–23]. Therefore we hypothesized that smoking may affect *CYP1A1* expression in human prostate tissue through the alteration of XRE CpG methylation in the enhancer region of this gene.

In this study, we assessed whether *CYP1A1* levels were elevated in prostate cancer compared to normal prostate or benign prostatic hyperplasia (BPH) using tissue microarray (TMA) of human specimens as well as prostatic cell lines (cancer versus BPH-1). Also, we evaluated the methylation level of XRE sites of the *CYP1A1* enhancer in cell lines and clinical samples and determined the effects of smoker status. Finally, we knocked the *CYP1A1* gene down in prostate cancer cell lines by RNA interference and performed functional analysis to evaluate its biological role in tumorigenesis.

## RESULTS

### *CYP1A1* expression in prostate cell lines and clinical samples

**Figure 1:**
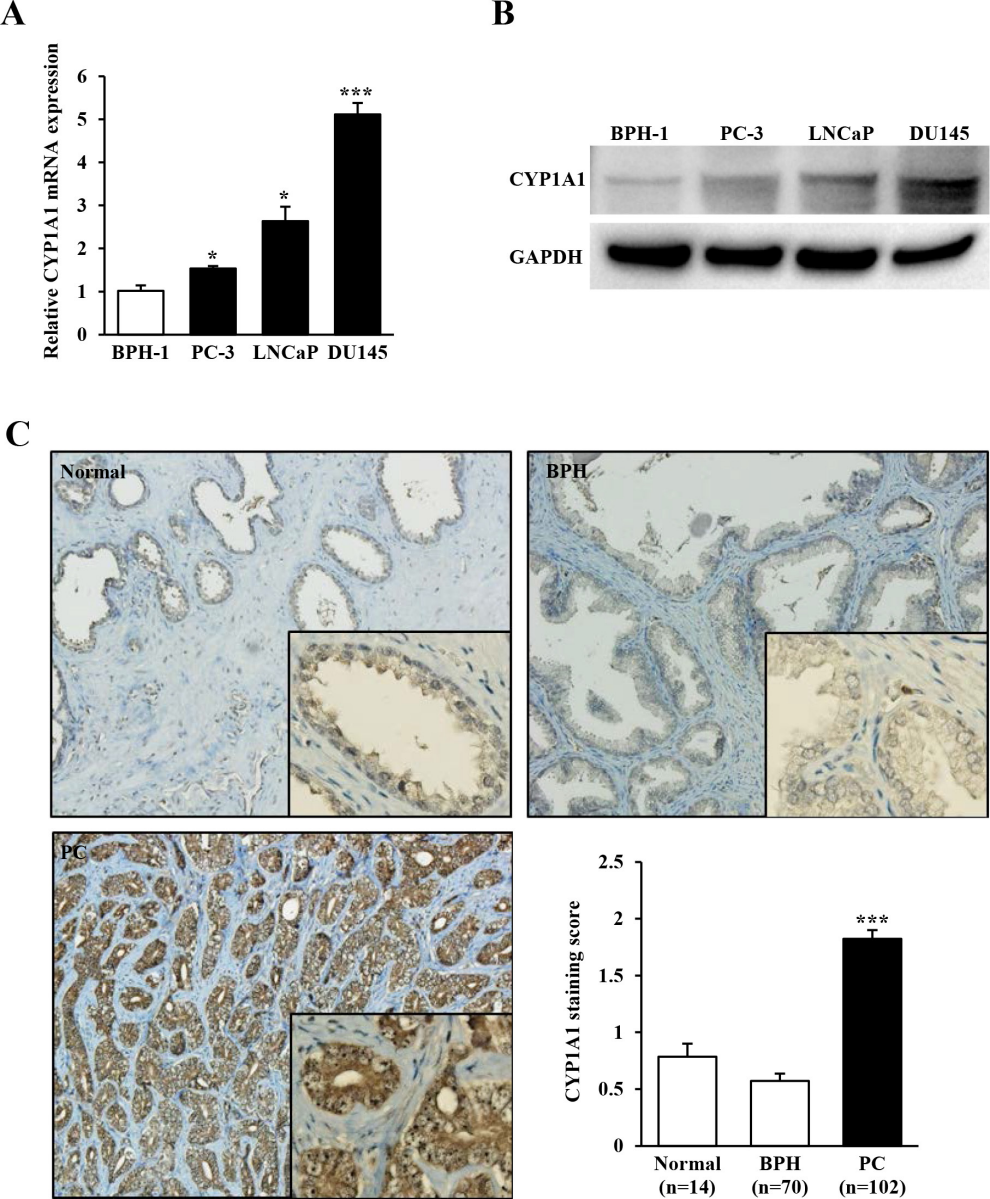
*CYP1A1* expression in prostate cancer cell lines and tissues (**A**) Relative mRNA expression levels of *CYP1A1* in prostate cancer cell lines (PC-3, LNCaP, and DU145) and benign cells (BPH-1) as measured by real-time PCR. The expression level of *CYP1A1* mRNA was significantly up-regulated in prostate cancer cell lines. Experiments done in triplicate; *P < 0.05, ***P < 0.001. (**B**) Representative immunoblot displaying *CYP1A1* (top gel) and *GAPDH* (bottom gel) in BPH-1, PC-3, LNCaP and DU145. (**C**) Representative immunostaining of *CYP1A1* in clinical samples (normal prostate, benign prostatic hyperplasia (BPH), and prostate cancer (PC) obtained from tissue microarray. Bar graph: *CYP1A1* protein expression in prostate cancer (*n* = 102) samples was significantly higher than that of normal (*n* = 14) or BPH (*n* = 70) tissues. ***P < 0.001.

Initially we measured mRNA and protein expression levels of *CYP1A1* in 3 prostate cancer cell lines (PC-3, LNCaP and DU145) and as a comparison, measured expression in BPH-1 cells. Both mRNA (Figure 1A) and protein (Figure 1B) were up-regulated with variable increases in cancerous cells with DU145 showing the largest elevation of expression compared with nonmalignant BPH-1 cells. Next we investigated the expression of *CYP1A1* by immunohistochemical staining in 102 primary prostate cancers, 14 normal prostate and 70 BPH samples obtained from TMAs. While *CYP1A1* expression was weak or not detected in most of the normal prostate (0.79 ± 0.11) and BPH (0.57 ± 0.07) tissues, the majority of prostate cancer samples showed much higher *CYP1A1* immunoreactivity with an average staining score of 1.82 ± 0.08 (*P* < 0.001, Figure 1C). Thus *CYP1A1* is up-regulated in prostate cancer cell lines and tissues.

### Increased expression of *CYP1A1* in prostate cancer cell lines after 5-aza-dC treatment

**Figure 2:**
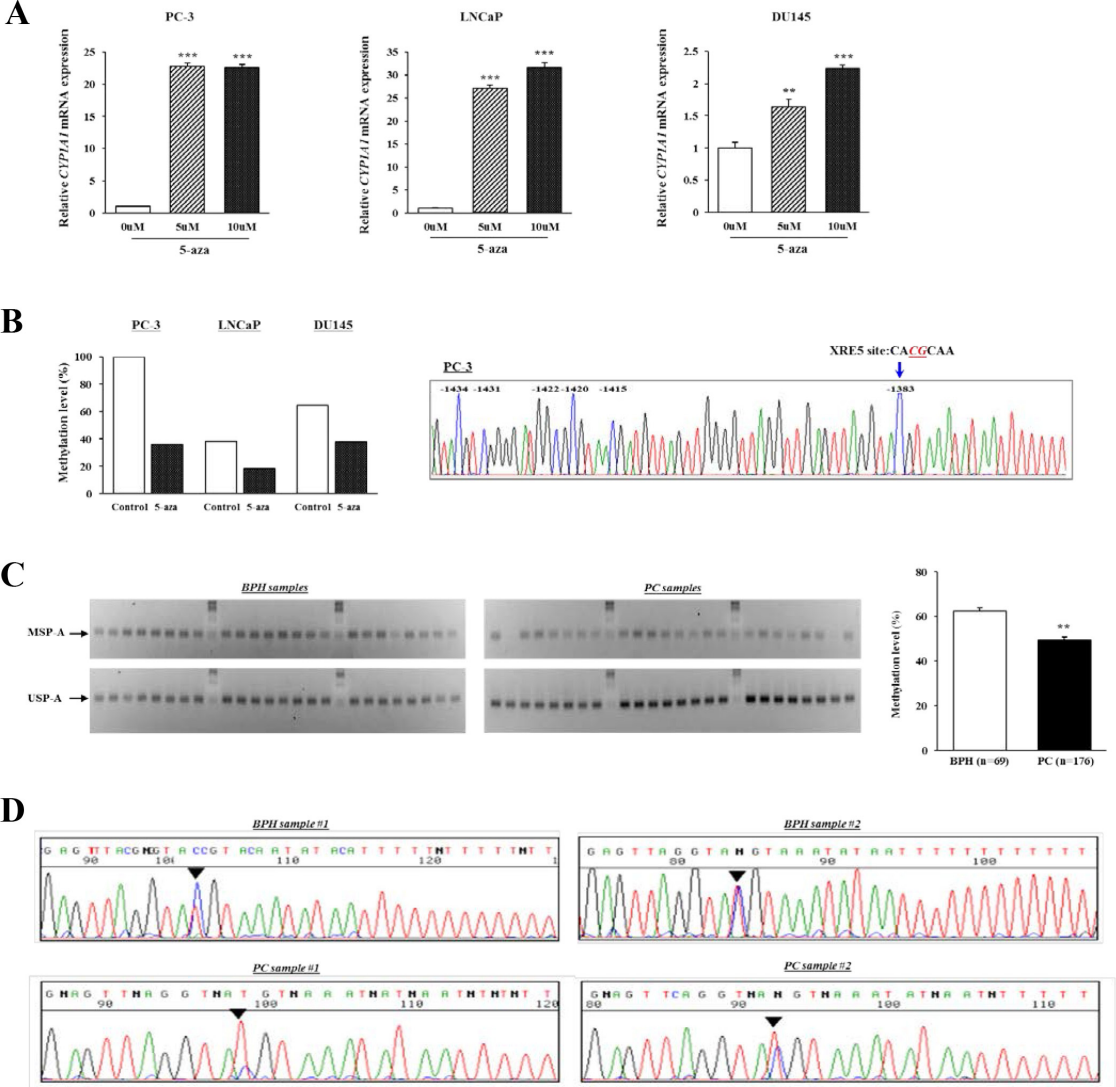
Analysis of *CYP1A1* methylation in region A (XRE-1383) (**A**) *CYP1A1* expression before and after demethylation (5-aza-dC) treatment in human prostate cancer cell lines as measured by real-time PCR. *CYP1A1* mRNA expression was significantly increased after 5-aza-dC treatment as compared with that before demethylation in PC-3, LNCaP, and DU145. Experiments done in triplicate; **P < 0.01, ***P < 0.001. (**B**) After 5-aza-dC treatment and performing methylation-specific PCR (MSP) on bisulfite-modified DNA, densitometry of MSP-A and USP-A bands were measured using Image J software and methylation calculated as MSP/[MSP+USP]. The amount of methylation dramatically decreased in all prostate cancer cell lines compared with untreated controls. Chromatogram: DNA sequencing shows CpG sites around region A were completely methylated in PC-3 cells. (**C**) Representative results of MSP-A and USP-A of *CYP1A1* enhancer in benign prostate hyperplasia (BPH) and prostate cancer (PC) samples. Bisulfite-modified DNA of clinical specimens underwent MSP. Top and bottom gels show MSP-A and USP-A bands, respectively. Each column is samples from the same patient. Bar graph: Methylation of *CYP1A1* was significantly lower in prostate cancer (PC, *n* = 176) than BPH (*n* = 69) samples as measured by densitometry of gel bands. **P < 0.01. (**D**) Typical bisulfite DNA sequencing chromatograms in BPH and prostate cancer (PC) samples. Though the region A site was partially methylated in samples, methylation was more prominent in BPH than in prostate cancer.

**Figure 6:**
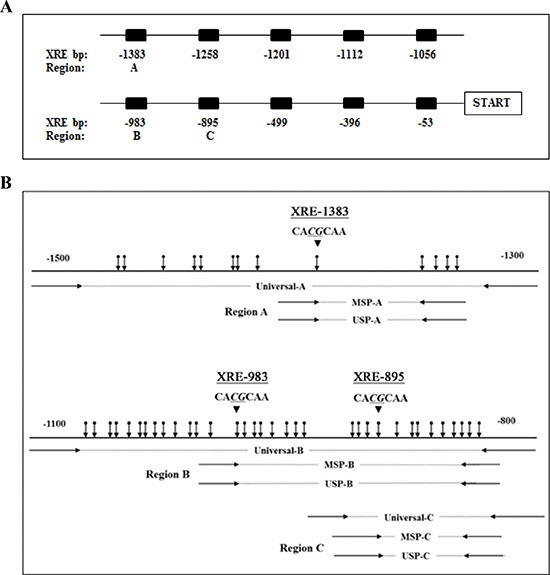
Diagram of the *CYP1A1* enhancers and locations of primers (**A**) Schematic representation of 10 xenobiotic responsive elements (XRE) located upstream of the transcription start site. Numbers represent bp prior to start site for each XRE. DNA methylation levels of *CYP1A1* enhancer region A (-1383 bp), B (-983 bp) and C (-895 bp) were evaluated in this study. (**B**) Location of designed primers utilized for amplification of regions A, B and C of the *CYP1A1* enhancer are depicted. Methylation-specific PCR (MSP) and unmethylation-specific PCR (USP) primers contain several CpG sites within the primer sequence whereas universal primers do not contain any CpG sites. Vertical arrows indicate CpG sites and large arrowheads indicate XRE core sequence.

We utilized 5-aza-deoxycytidine (5-aza-dC) to screen the methylation status of *CYP1A1* in prostate cancer cell lines. As shown in Figure 2A, *CYP1A1* mRNA expression was markedly increased in all cancer cells after treatment indicating that promoter CpG methylation affects *CYP1A1* expression. To validate the relationship between CpG methylation and expression of the *CYP1A1* transcript, we performed methylation-specific PCR (MSP) analysis of region A (MSP-A) of the enhancer region which contains the XRE-1383 site (see Figure 6A, 6B) using bisulfite-modified DNA of treatment groups. In control cells, densitometry measurements of MSP-A and USP-A bands show higher levels of methylation in all cancer lines with PC-3 having 100% (Figure 2B). Bisulfite DNA sequencing confirmed that most CpG sites in region A area were completely methylated in untreated PC-3 cells (Figure 2B). After 5-aza-dC treatment however, a dramatic reduction in % methylation of this site were observed in all cancer lines. These results show that expression of *CYP1A1* is inversely regulated by methylation of region A of the promoter in human prostate cancer cell lines.

### Methylation level of the *CYP1A1* enhancers in prostate tissues

Since Figure 1C indicated increased expression of *CYP1A1* in prostate cancer regions, we then determined whether cancer tissues also have lower methylation levels. MSP of region A in 176 prostate cancer and 69 BPH samples were analyzed. Representative MSP-A and USP-A bands of 24 BPH and 24 prostate cancer tissues are shown in Figure 2C. The relative methylation amount of the *CYP1A1* promoter in region A was significantly lower in prostate cancer (49.2 ± 1.6%) than in BPH samples (62.5 ± 1.3%; *P* < 0.01). Also, bisulfite DNA sequencing was performed to confirm whether the MSP bands reflect the true methylation status of the *CYP1A1* enhancer. Representative sequence chromatograms of two BPH and two prostate cancer samples in region A are shown in Figure 2D and methylation of the XRE-1383 site was generally higher in BPH than in prostate cancer tissues. These results indicate that methylation analysis by MSP was consistent with bisulfite DNA sequencing.

**Figure 3:**
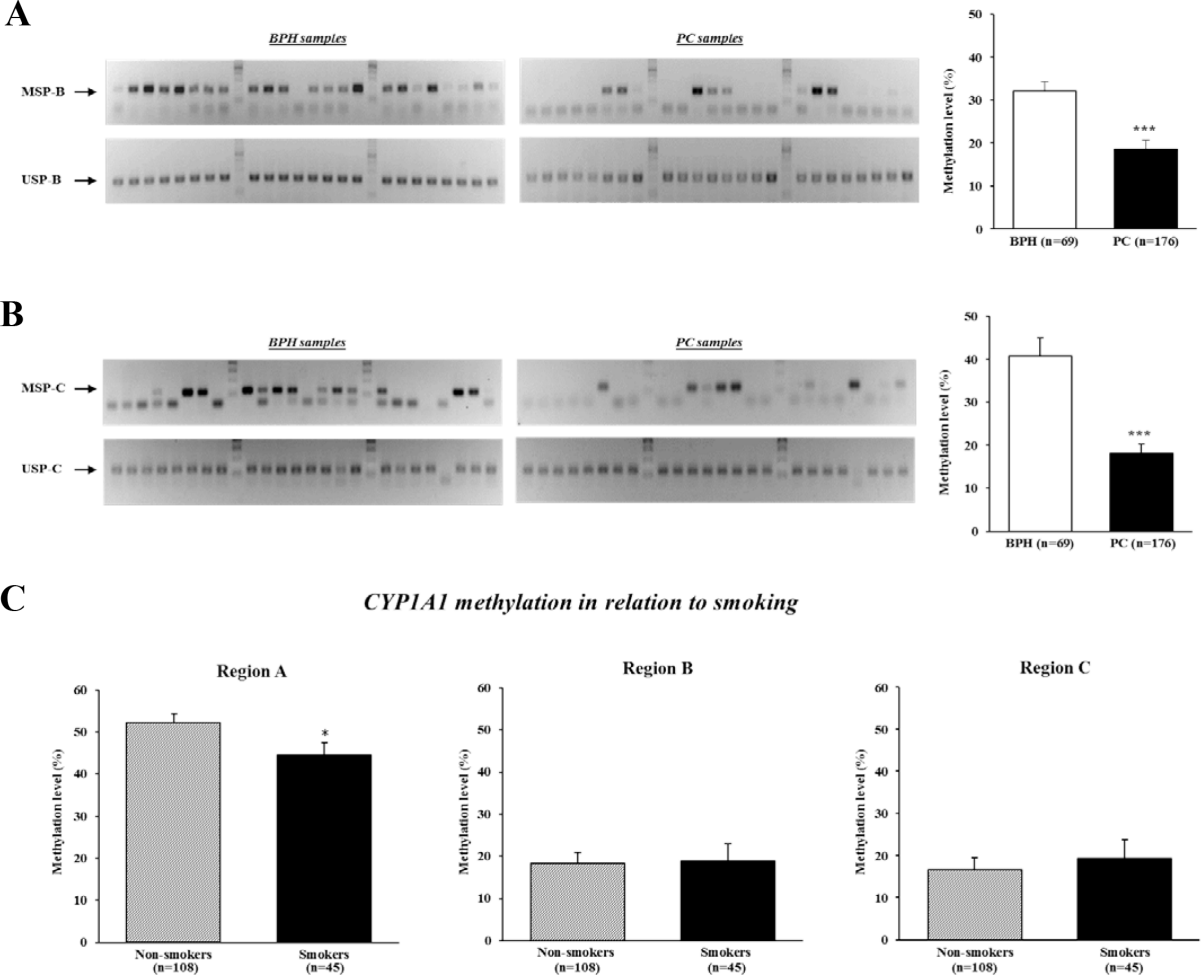
Analysis of *CYP1A1* methylation in enhancer regions B (XRE-983) and C (XRE-895), and effect of smoking on methylation levels DNA of clinical specimens underwent bisulfite modification followed by methylation-specific PCR (MSP) analyses. (**A**) Representative gel results of MSP-B (top gel) and unmethylation-specific PCR (USP)-B (bottom gel) of *CYP1A1* enhancer in benign prostate hyperplasia (BPH) and prostate cancer (PC) samples. Bar graph: The prevalence of *CYP1A1* methylation was significantly lower in prostate cancer (*n* = 176) than BPH (*n* = 69) samples as measured by densitometry of gel bands. ***P < 0.001. (**B**) Representative gel results of MSP-C (top gel) and USP-C (bottom gel) of *CYP1A1* enhancer in BPH and prostate cancer (PC) samples. Bar graph: A significant reduction in *CYP1A1* methylation levels in prostate cancer (PC) samples were also observed for this site. ***P < 0.001. (**C**) *CYP1A1* methylation levels in relation to tobacco smoker status at all 3 regions. Smoking habit affected the methylation level of *CYP1A1* region A. Non-smokers *n* = 108, Smokers *n* = 45; *P < 0.05.

MSP analysis of other *CYP1A1* enhancers containing XRE-983 (region B) and XRE-895 (region C) (see Figure 6A, 6B) was also performed. As shown in Figure 3A, most BPH tissues showed both MSP-B and USP-B bands whereas many prostate cancer tissues had only a USP-B band. The relative methylation level of *CYP1A1* in region B was significantly lower in prostate cancer (18.5 ± 2.1%) than BPH samples (32.1 ± 2.0%; *P* < 0.001). Likewise, prostate cancer tissues have lower *CYP1A1* methylation in region C in comparison with BPH samples (18.0 ± 2.3% and 40.7 ± 4.2%, respectively; *P* < 0.001, Figure 3B). Therefore DNA hypomethylation of three *CYP1A1* XRE sites was more common in prostate cancer than BPH tissues. We further evaluated the relationship between the methylation level of the *CYP1A1* enhancer and pathological status; however, no significant association was found for either grade or stage of cancer (data not shown).

### Methylation of *CYP1A1* enhancers in smoker and non-smoker prostate cancer tissues

We then evaluated the association between smoker status and the methylation level of *CYP1A1* enhancers in prostate cancer patients. Of 176 patients who underwent radical prostatectomy, 78 never smoked, 30 were ex-smokers who had quit smoking 7 or more days before surgery, 45 were current smokers, and the remaining 23 were unknown. A past history of smoking was not significantly associated with the methylation level in each region (data not shown). Interestingly however, the methylation level of *CYP1A1* in the region A site was significantly higher in 108 nonsmokers (never smokers and ex-smokers for > 7 days combined) than 45 smokers (52.2 ± 2.1% and 44.6 ± 2.9%, respectively; *P* < 0.05, Figure 3C). Cohen's d value for this difference is calculated to be 0.36 which is moderate. No differences were found in regions B and C. Thus tobacco smoking may affect the methylation of the *CYP1A1* enhancer region A.

### Effect of *CYP1A1* knockdown on prostate cancer cell growth

**Figure 4:**
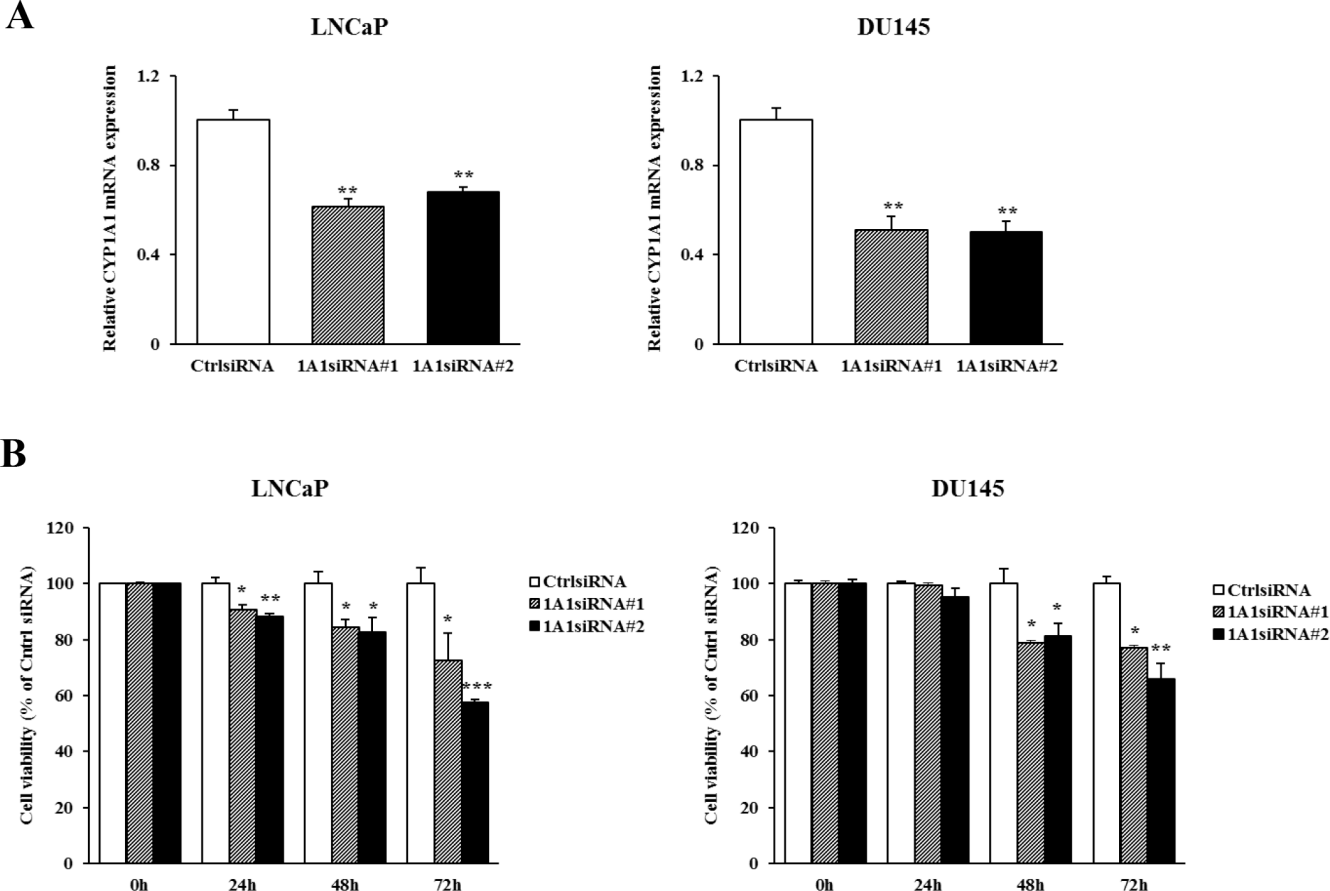
Effect of *CYP1A1* knockdown on cell proliferation in prostate cancer cell lines (**A**) Knockdown of *CYP1A1* levels in LNCaP and DU145 were determined by real-time PCR at 48 hours after transfection with two different *CYP1A1* siRNAs. Both siRNA's significantly reduced *CYP1A1* expression. Experiments done in triplicate; **P < 0.01. (**B**) Cell viability was analyzed by the MTS cell proliferation assay at 0, 24, 48 and 72 hours after siRNA treatment. In both cell lines, the attenuation of *CYP1A1* significantly inhibited cell viability in a time dependent manner. Experiments done in triplicate; *P < 0.05, **P < 0.01, ***P < 0.001.

**Figure 5:**
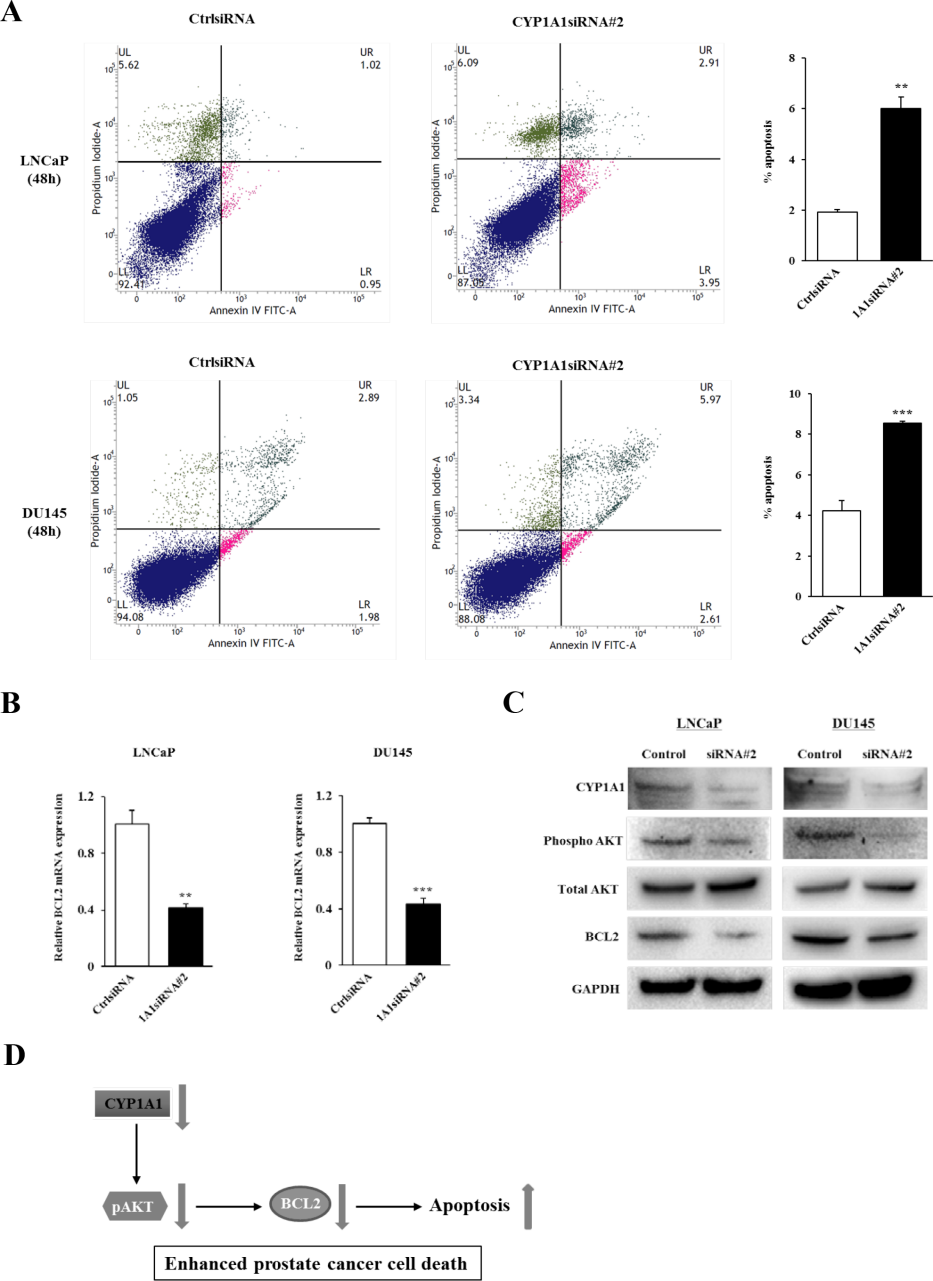
Effect of *CYP1A1* knockdown on apoptosis (**A**) Apoptosis assays with LNCaP (upper graphs) and DU145 (lower graphs) cells were measured 48 hours post-siRNA#2 transfection by flow cytometry. Representative biparametric histograms showing cell populations in early (bottom right quadrant) and late (top right quadrant) apoptotic, and viable (bottom left quadrant) states for each treatment. Bar graph: The percentage of apoptotic cell fractions (early plus late apoptotic cells) in *CYP1A1* siRNA#2 transfectants were significantly elevated compared to controls. Experiments done in triplicate; **P < 0.01, ***P < 0.001. (**B**) Verification of cDNA microarray data. Among 5 genes significantly altered due to siRNA#2 (see Supplementary Table S1), only *BCL2* was confirmed to be down-regulated by real-time PCR using Taqman probe in both LNCaP and DU145. No differences in remaining genes were observed. Experiments done in triplicate; **P < 0.01, ***P < 0.001. (**C**) Immunoblotting analysis of *CYP1A1*, phospho *AKT*, total *AKT* and *BCL2* in control and *CYP1A1* siRNA#2-transfected LNCaP and DU145 cells. *GAPDH* was used as a loading control. (**D**) Schematic representation of the proposed effect of *CYP1A1* down-regulation in prostate cancer. *CYP1A1* knockdown inhibits *BCL2* expression through the inactivation of *AKT*, leading to prostate cancer cell death.

The higher basal expression levels of *CYP1A1* in prostate cancer tissues led us to examine the functional significance of *CYP1A1* in prostate cancer. We determined this by testing the effects of *CYP1A1* reduction on prostate cancer cell viability. LNCaP and DU145 were selected as these showed higher constitutive levels of *CYP1A1* (Figure 1A and 1B). After transfection with two different *CYP1A1* siRNAs (#1 and #2), significant reduction of *CYP1A1* mRNA was found in both LNCaP and DU145 cells (Figure 4A). *CYP1A1* protein was also lowered as well (see Figure 5C). As shown in Figure 4B, knockdown of *CYP1A1* caused the growth of these cells to be significantly inhibited in a time-dependent manner as compared with controls.

### Effect of *CYP1A1* knockdown on apoptosis

Since attenuation of *CYP1A1* significantly inhibited proliferation of prostate cancer cell lines, we hypothesized that this may be due to apoptosis induction. Apoptosis was examined in control siRNA and *CYP1A1* siRNA#2-treated cells and results for LNCaP and DU145 cells at 48 hours post-transfection are shown in Figure 5A. In both cell lines, the apoptotic and early apoptotic fractions (upper right and lower right quadrants, respectively) were significantly greater in *CYP1A1*-depleted cells compared to control cells. Similar results were also observed in both LNCaP and DU145 cells at 72 hours after siRNA transfection (data not shown). This supports an anti-apoptotic role for *CYP1A1* suggesting that it regulates tumorigenicity by affecting the apoptotic pathway.

To further understand the precise mechanism of the pro-apoptotic effect on prostate cancer cells induced by *CYP1A1* knockdown, we performed array analyses to determine which apoptotic genes are altered due to *CYP1A1* in LNCaP cells. There were 3 genes up-regulated and 2 genes down-regulated two-fold or greater after *CYP1A1* depletion (Supplementary Table S1). Among these 5 genes, only *BCL2* was found to be significantly altered in both LNCaP and DU145 cells by real-time PCR using Taqman probes (Figure 5B). Also, *BCL2* protein levels were significantly decreased in *CYP1A1* siRNA#2-treated cells compared with control in both cell lines (Figure 5C). These data indicate that attenuation of *CYP1A1* expression caused reduction of *BCL2* expression with increased apoptotic effects in prostate cancer. Furthermore, we sought to determine whether *CYP1A1* knockdown inhibits *AKT* phosphorylation since a recent study showed that *CYP1A1* is associated with the *PI3K-AKT* signaling pathway in breast cancer cell lines [8]. As shown in Figure 5C, *CYP1A1* knockdown reduced *AKT* phosphorylation in both cell lines indicating *CYP1A1* may be an upstream activator of the *PI3K-AKT* signaling pathway. Figure 5D describes a proposed simplified schematic representation of the effect of *CYP1A1* down-regulation in prostate cancer cells.

## DISCUSSION

In this study, we demonstrate that prostate cancer has high expression of *CYP1A1* through DNA hypomethylation of XRE sites. It is well known that methylation of CpG sites within the gene promoter is significantly related to down-regulation or silencing of several types of genes. Previous studies have shown that the expression of human *CYP1A1* gene may, at least in part, be regulated by methylation of the XRE core sequence 5′-GCGTG-3′ (or 5′-CACGC-3′) located in the 5′-upstream region [16–19]. Our study on prostate cancer also supports regulation by methylation as treatment of PC-3, LNCaP, and DU145 with demethylating agent caused an enhanced increase of *CYP1A1* expression with dramatic loss of methylation as shown by MSP/USP analyses of the enhancer XRE-1383 site. These results are in agreement with Sterling and Cutroneo [24] who find *CYP1A1* protein to be expressed constitutively in all of these cancer lines; and Okino et al [16]. observes LNCaP to be methylated at enhancer sites of the *CYP1A1* promoter. It is worthy to note that despite the presence of methylation at the XRE-1383 site, all untreated cancer cells displayed increased though variable constitutive levels of *CYP1A1* (compared to BPH-1). However paradoxical, we can point out that PC-3 cells that showed complete methylation by MSP/USP densitometry and bisulfite sequencing had only a small increase in *CYP1A1* expression, whereas LNCaP and DU145 displayed partial methylation that can lead to relatively higher gene expression. Compared to LNCaP, it is not known why DU145 had increased *CYP1A1* expression despite elevated levels of methylation. Other epigenetic processes such as histone acetylation and deacetylation has been shown to affect gene transcription [25] and further studies are needed to determine if this could also play a role. Nonetheless, 5-aza-dC treatment showed a clear and pronounced inverse effect between expression and methylation of *CYP1A1*.

In tissue, a similar pattern was also observed as TMA specimens displayed a significant increase of *CYP1A1* expression in prostate cancer compared to normal and BPH; and methylation analyses of clinical specimens exhibited cancer to be hypomethylated at all three enhancer sites (XRE-1383, XRE-983, XRE-895) compared to BPH. In contrast to our results, Okino et al. [16] did not detect methylation in the enhancer region of all 30 non-cancerous prostates whereas 11 of 30 tumor specimens showed methylation. Reasons for discrepancy with our study are not known but in their study, expression of *CYP1A1* was not measured and would be of interest if a correlation existed with methylation in their clinical specimens. In support of our results however, in other studies that utilized normal prostate tissues only, Martin et al. [26] determined that all 10 specimens were devoid of *CYP1A1* by Western analysis and John et al. [7] observed minimal gene expression by real-time PCR. Thus taken together with our results of prostatic tissues and cell lines, these findings strongly suggest that DNA hypomethylation of the *CYP1A1* enhancer is a frequent event in prostate cancer and involved in the induction of *CYP1A1* expression.

Recently several epidemiologic reports using relatively large cohorts suggest that tobacco smoking, an important risk factor for several cancers [27, 28], may also be associated with prostate cancer [20–23]. It is postulated that the induction of several carcinogen relevant genes, especially *CYP1A1*, could play important roles in smoking-induced tumorigenesis [29, 30]. In the prostate, studies by Sterling and Cutroneo [24] and Hruba et al. [6] showed that *CYP1A1* expression was enhanced by benzo[a]pyrene, a genotoxin in tobacco smoke. These reports led us to determine the possible association between smoking and methylation levels of the *CYP1A1* enhancer in prostate cancer patients. Here, we clearly show for the first time that methylation of the *CYP1A1* enhancer encompassing the XRE-1383 site was especially affected by smoking with a medium effect size difference as measured by Cohen's d value; and this observation is in agreement with previous experiments on human lung tissues [17, 18]. Though we haven't measured the correlation of CYP1A1 expression in tissue with smoker status, the evidence is strong to predict that CYP1A1 levels would be higher in smokers due to lower methylation. Thus smoking could influence the pathogenesis of prostate cancer via the epigenetic dysregulation of *CYP1A1*. The putative mechanisms underlying tobacco smoking and regulation of *CYP1A1* methylation is that smoking promotes AhR/ARNT heterodimer binding to XREs which leads to loss of methylation by eliminating DNA methyltransferases from the *CYP1A1* enhancer [19]. Therefore tobacco smoke may indirectly enhance demethylation of the *CYP1A1* enhancer.

It should be noted that the expression level of many genes induced by smoking can be reduced after smoking cessation [31]. In the case of *CYP1A1*, a study by Anttila et al. [17] showed the methylation level of its promoter to increase within 1–7 days after quitting smoking in lung cancer which may inhibit the binding of AhR/ARNT heterodimer and subsequently lead to a reduction of *CYP1A1* expression. In our study, ex-smokers who had quit smoking 7 or more days before surgery combined with never-smokers showed higher *CYP1A1* methylation levels compared with current smokers, indicating smoking to be a modifiable risk factor for prostate cancer that is dependent on *CYP1A1* expression. This observation can help to add to the recommendation for tobacco smoking cessation.

Significant up-regulation of *CYP1A1* has been observed in several types of malignancies, i.e. esophagus, breast, urinary bladder, and brain tumor, and is associated with poor prognosis [32–36]. These findings are conceivable since *CYP1A1* is known to play an important role in the formation of PAH-DNA adducts which may induce mutations in several cancer-related genes that contribute to carcinogenesis [37, 38]. Though previous research has failed to demonstrate a role for PAH-DNA adducts as a cancer factor in the prostate [6, 7], our results showing that prostate cancer has high *CYP1A1* expression as compared to normal/benign prostate tissues suggests that *CYP1A1* may contribute to prostate cancer pathogenesis. We observe that reduction of basal *CYP1A1* expression leads to significant inhibition of cell proliferation through induction of apoptosis in prostate cancer cells, indicating *CYP1A1* expression is critical for cell growth, and this is in concordance with Hruba et al. [6] who showed that enhanced *CYP1A1* levels induced by benzo[a]pyrene displayed no effect on either apoptosis or cell cycle arrest in LNCaP cells. Interestingly, a recent study has also revealed that the attenuation of *CYP1A1* could prevent breast cancer progression even in the absence of xenobiotics [8]. Thus *CYP1A1* appears to have both canonical carcinogen metabolic function as well as its own oncogenic function.

We further identified that the effects of *CYP1A1* knockdown on increased apoptosis in prostate cancer cell lines were correlated with reduction of anti-apoptotic *BCL2*, the primary gatekeeper of the intrinsic (mitochondrial) apoptotic pathway. This is expected since metabolites of *CYP1A1* such as heterocyclic aromatic amines have been shown to cause increase in *BCL2* mRNA and protein [39]. *BCL2* is commonly overexpressed in prostate cancer compared to normal prostate [40] and elevation of *BCL2* expression is associated with poor prognosis and suspected in the development of castration-resistant prostate cancer [41–43]. Currently several clinical trials targeting *BCL2* expression in prostate cancer are ongoing [44]; therefore *CYP1A1* may be a promising target for prostate cancer treatment since it is closely associated with *BCL2* expression. What remain unclear are the mechanisms of *CYP1A1* regulation of *BCL2*. Expression of *BCL2* is involved in the *PI3K-AKT* signaling pathway implicated in the development and progression of prostate cancer [45–47] and indeed, *CYP1A1* metabolites have been shown to lead to *AKT* phosphorylation [48]. *AKT* activated by *PI3K* leads to the activation of *NFkB*, which then translocates to the nucleus and transcribes *BCL2*. In this study, we show that *CYP1A1* knockdown can cause reduction of *AKT* phosphorylation in prostate cancer cells and this effect was also shown in breast cancer cells [8]. This inhibition of *AKT* could then promote *FOXO3a* and *Par-4* activation leading to apoptosis as was demonstrated in prostate cancer cells by Das et al. [49]. Also in a report by Zhu et al. [50], silencing of prostatic PC3 cells for *AKT* resulted in reductions of growth promoting *RANKL*, *PTHrP*, and *BMP-2*. Taken together, our results suggest that *CYP1A1* may act as an upstream activator of the *PI3K-AKT* signaling pathway and enhance the transcriptional activation of anti-apoptotic genes that include *BCL2*; and further studies are needed to verify this hypothesis.

In summary, we show that *CYP1A1* is an important oncogene in prostate cancer that is up-regulated by DNA promoter hypomethylation. Tobacco smoking can cause a further reduction of methylation at the *CYP1A1* distal enhancer that may lead to carcinogen activation through increased *CYP1A1* expression. Furthermore, *CYP1A1* is shown to have its own oncogenic function and promote prostate cancer proliferation and survival via dysregulation of *BCL2*. Altogether our findings demonstrate that tobacco smoking may contribute to the pathogenesis of prostate cancer and that inhibition of *CYP1A1* may have therapeutic potential.

## MATERIALS AND METHODS

### Tissue microarray

In total, 102 primary prostate cancer tissues composed of 10 specimens from TMA PR804, 36 from TMA PR956, and 56 from TMA PR208 (all acquired from US Biomax, Rockville, MD) were evaluated. Also, 14 normal prostate and 70 BPH specimens were included in these 3 TMAs.

### Clinical samples

A total of 176 newly diagnosed prostate cancer tissues from radical prostatectomy and 69 pathologically proven BPH samples from transurethral resection were obtained from a urology tissue bank at the Veterans Affairs (VA) Medical Center. The pathological background of the prostate cancer patients includes Gleason < 4 – 33 cases, Gleason 5 – 33 cases, Gleason 6 – 31 cases, Gleason 7 – 47 cases and Gleason > 8 – 32 cases; pT2 – 117 cases, pT3 – 56 cases and pT4 – 3 cases. The median age (range) of prostate cancer and BPH patients were 69 (49–80 years) and 75 (54–87 years), respectively. Our routine strategy to diagnose prostate cancer included serum PSA level, transrectal ultrasonography, color Doppler ultrasonography [51] and MRI that enabled us to accurately localize prostate cancer before radical prostatectomy. A portion of each tissue sample was fixed in 10% buffered formalin (pH 7.0) and embedded in paraffin wax. Sections (5 μm) were used for hematoxylin and eosin staining for histological evaluation. Another portion of tissue was frozen fresh and stored at −80°C until analyzed. Written informed consent was obtained from each patient for molecular analyses of the resected specimens and this study was approved by the Clinical Research Office of the San Francisco VA Medical Center and the Institutional Review Board of the University of California at San Francisco.

### Cell lines and reagents

Human prostate cancer cell lines (PC-3, LNCaP, and DU145) were purchased from the American Type Culture Collection (Manassas, VA). Keratinocyte serum-free medium, bovine pituitary extract and human recombinant epidermal growth factor were purchased from Invitrogen (Carlsbad, CA). RPMI 1640, Opti-minimum essential medium and penicillin/streptomycin were obtained from the UCSF Cell Culture Facility (San Francisco, CA). Fetal bovine serum was a product of Atlanta Biologicals (Lawrenceville, GA). All cell lines were cultured in RPMI 1640 medium supplemented with 10% FBS. Cells were maintained in a humidified atmosphere of 5% CO_2_/95% air at 37°C.

### Nucleic acid extraction

Genomic DNA from prostate cancer and BPH tissue samples were extracted using a DNA extraction kit (Qiagen, Valencia, CA). Genomic DNA from cell line samples were extracted using DNAzol reagent (Life Technologies, San Diego, CA) and total RNA was extracted with TRI reagent (Molecular Research Center, Cincinnati, OH) according to the manufacturer's instructions. The RNA pellet obtained after isopropanol and ethanol precipitation was dried, resuspended in RNase-free water, and stored in aliquots of 25 μL at −80°C until reverse-transcribed. The concentrations of DNA and RNA were determined with a spectrophotometer and their integrity was checked by gel electrophoresis.

### cDNA preparation and gene quantification

Using 1 μg of RNA, 0.5 μg of oligo-dT primer and 0.5 units of RNase inhibitor, cDNA was constructed using reverse transcriptase (Promega, Madison, WI). The mRNA transcript levels of *CYP1A1*, *CD70*, *MCL1*, *TNFRSF11B*, *FAS* and *BCL2* were measured by the 7500 Fast Real-Time PCR System (Applied BioSystems, Foster City, CA) with *GAPDH* used as the reference gene. The data were analyzed by the delta-delta Ct method to calculate the fold-change.

### Western blot analysis

Whole cell extracts from cell lines were prepared using radio-immunoprecipitation assay buffer (RIPA; Thermo Scientific, Rockford, IL) containing protease inhibitor cocktail (Roche Diagnostics, Basel, Switzerland). Protein quantification was done using a BCA protein assay kit (Thermo Scientific) according to the manufacturer's instructions. Total cell protein (15–20 μg) was used for Western blotting. Samples were transferred to PVDF membranes that were immersed in 3% skim milk containing antibody against *CYP1A1* (polyclonal #ab3568, Abcam, Cambridge, MA), *AKT* (monoclonal #4691, Cell Signaling Technology, Danvers, MA), phospho-*AKT* (monoclonal #4060, Cell Signaling Technology), *BCL2* (polyclonal sc-492, Santa Cruz Biotechnology, Dallas, Texas), and *GAPDH* (monoclonal sc-47724, Santa Cruz Biotechnology) overnight at 4°C. Blots were washed in TBS containing 0.1% Tween20 and labeled with horseradish peroxidase conjugated, secondary anti-rabbit antibody (Cell Signaling Technology). Specific complexes were visualized with an enhanced chemiluminescence detection system (GE Healthcare, Little Chalfont, UK) using the Chemidoc imaging system (Bio-Rad, Hercules, CA). *GAPDH* was used as control for equal protein loading.

### 5′-Aza-2-deoxycytidine (5-aza-dC) treatment

Prostate cancer cell lines were treated with 5-aza-dC (Sigma-Aldrich, St Louis, MO) to screen for epigenetic alterations. 5-aza-dC was added to fresh cell culture medium at a concentration of 5 μM in duplicate wells. The cultured cells were harvested after 4 days of treatment. The mRNA transcripts before and after 5-aza-dC treatment were analyzed by real-time PCR after cDNA conversion.

### Bisulfite DNA sequencing and methylation analysis

Genomic DNA (100 ng) was modified with sodium bisulfite (Sigma-Aldrich) using a commercial kit (Life Technologies). In the *CYP1A1* promoter, up to 10 high affinity binding sites for the AHR complex has been identified [52] and are shown in Figure 6A. Based on previous findings the methylation of the −1383 bp CpG site located within the distal XRE was significantly affected by smoking [17, 18] and shown to regulate *CYP1A1* expression [18], and our methylation analysis was focused on this CpG site (XRE-1383, region A, Figure 6A). In addition, we evaluated region B which contains XRE at −983 bp (XRE-983) that also is affected by smoking [18] as well as region C that contains XRE at the −895 bp site (XRE-895, Figure 6A). Universal, methylation-, and unmethylation-specific PCR primers were designed using a MethPrimer program (http://itsa.ucsf.edu/~urolab/methprimer) according to our previous studies [12–14]. The 3 regions (A, B, and C) amplified by these primers have 5, 22 and 13 CpG sites, respectively, and a schematic diagram of the location of these primers and CpG sites are shown in Figure 6B. For MSP (methylation-specific PCR), a second round of nested PCR (MSP and USP (unmethylation-specific PCR)) was done using the universal PCR product amplified by universal-sense and universal-antisense primers as a template. The universal primer sets contain no CpG sites in either the forward or reverse primer. In each amplification, the absence of a DNA template served as a negative control. The universal primer sequences for regions A through C were 5′-GGGATTATTTTTTGGTTTGGATTA-3′ (sense) and 5′-CATAACCTAACTACCTACCTCC-3′ (antisense), 5′-AGGTTGGTTTTTTAAGAGTTT-3′ (sense) and 5′-ATTAACAAAACACAAAAATCC-3′ (antisense), and 5′-TTTGTTTTTTAGAGGGATGT-3′ (sense) and 5′-CTTTAATTAACAAAACACAAAAAT-3′ (antisense), respectively. The MSP primer sequences for regions A-C were 5′- GGGTTAGGTGAGTTAGGTCG-3′ (sense) and 5′- CCGACGCTATCCCGCCCTCCG-3′ (antisense), 5′- CGGGTTTTCGGTTTTTTTTAC-3′ (sense) and 5′- CCCGACTCTAACTTACGTACG-3′ (antisense), and 5′- TAGAGGGATGTCGTCGGCGTAC-3′ (sense) and 5′- TAACAAAACACAAAAATCCGACGACG-3′ (antisense), respectively. The USP primer sequences for regions A-C were 5′- TGGGGTTAGGTGAGTTAGGTTG-3′ (sense) and 5′- TCCAACACTATCCCACCCTCCA-3′ (antisense), 5′- GTGGGTTTTTGGTTTTTTTTATGT-3′ (sense) and 5′- CCCCAACTCTAACTTACATACACC-3′ (antisense), and 5′- TAGAGGGATGTTGTTGGTGTAT-3′ (sense) and 5′- TAACAAAACACAAAAATCCAACAACA-3′ (antisense), respectively. The MSP and USP products were analyzed by 2% agarose gel electrophoresis. Density of each band was calculated by Image J software (http://rsb.info.nih.gov/ij) and the relative methylation level in each sample was determined using the following formula; MSP ratio (%) = MSP /[MSP+USP]. DNA sequencing was also performed on bisulfite-modified DNA. One μl of modified DNA was amplified using a pair of universal primers in a total volume of 20 μL. Sequencing of the PCR products using either a forward or reverse universal primer was done according to the manufacturer's instructions (Applied Biosystems).

### Knockdown of *CYP1A1* in LNCaP and DU145 cells

siRNA oligonucleotides against human *CYP1A1* and mismatch control oligonucleotides were purchased from Life Technologies. For inhibition of *CYP1A1*, 5 μl of siRNA oligonucleotides (siRNA-*CYP1A1* (s3800 (designated #1), s3801 (designated #2), or siRNA-control) and 5 μl of lipofectamine RNAiMAX reagent (Life Technologies) were diluted with 250 μl of Opti-MEM (Gibco, Carlsbad, CA). Cells were then transfected with lipofectamine+siRNA-*CYP1A1* (#1 or #2) or siRNA-control. Transfection was terminated after 5 hours by aspirating the transfection medium including non-adherent cells and adding fresh RPMI 1640 containing 10% FBS. The remaining cells were incubated at 37°C.

### MTS assay

Cells were plated in triplicate in 96-well microplates at a density of 3 × 10^3^ cells per well. After treatment with *CYP1A1* siRNA, the number of viable cells was determined over time by adding 3-(4,5-dimethylthiazol-2-yl)-5-(3-carboxymethoxyphenyl)-2-(4-sulfophenyl)-2H-tetrazolium-based CellTiter 96 Aqueous One Solution Reagent (Promega) to each well and measuring the absorbance at 490 nm on SPECTRA MAX 190 plate reader (Molecular Devices, Sunnyvale, CA).

### Apoptosis assay

Fluorescence-activated cell-sorting (FACS) analysis for apoptosis was done 48 hours post-transfection using an annexin V-fluorescein isothiocyanate (FITC)/7-amino-actinomycin D (7-AAD) staining system obtained from BD Biosciences (San Jose, CA) and a BD FACSVerse^™^ flow cytometer (BD Biosciences). Cells were stained with annexin V-FITC only (early apoptotic) or both annexin V-FITC and 7-AAD (late apoptotic), and the combined sum was considered to be the total apoptotic cell fraction.

### Apoptosis-related gene array analyses

cDNAs from control and *CYP1A1* siRNA#2-treated cells were evaluated for gene expression using the RT^2^ Profiler^™^ PCR Array PAHS-012ZC (Human Apoptosis PCR Array, Qiagen) on the ABI Fast 7500 Real-Time PCR System with RT^2^ Real-Time SYBR Green PCR master mix according to the manufacturer's protocol.

### Immunohistochemical analyses

Immunostaining of *CYP1A1* was performed on TMA (tissue microarray) slides using the UltraVision Detection System (Thermo Scientific) according to the manufacturer's instructions. After 12 hours incubation with rabbit polyclonal antibody for *CYP1A1* (1:200, #ab3568, Abcam), 3,3′-diaminobenzidine was added as chromogen followed by counterstaining with hematoxylin. The degree of immunostaining was evaluated by two independent observers. Cytoplasmic expression was analyzed by the intensity of positive cells using Image J software (http://rsb.info.nih.gov/ij) and was ranked on an overall scale from 0 to 3; with 0 indicating the absence of staining; 1, weak staining; 2, moderate staining; and 3, strong staining according to our previous study [53].

### Statistical analyses

Values are presented as the mean ± standard error based on results obtained from at least three independent experiments. All data were analyzed using StatView 5 statistical software (SAS Institute, Inc., Cary, NC). The relationship between two variables was analyzed using the two-tailed unpaired Student's *t*-test. Cohen's d value was calculated to determine effect size difference between groups. A *p*-value of less than 0.05 was considered to be statistically significant.

## SUPPLEMENTARY MATERIALS TABLE


