# Supplementary material for: Functional role and tobacco smoking effects on methylation of CYP1A1 gene in prostate cancer
Source: Oncotarget. 2016 May 19;7(31):49107–21. doi: 10.18632/oncotarget.9470 (PMC5226494; doi:10.18632/oncotarget.9470)
Supplement: Supplementary file 1 [file oncotarget-07-49107-s001.pdf]

Functional role and tobacco smoking effects on methylation of *CYP1A1* gene in prostate cancer

Supplementary Materials

Supplementary Table S1: cDNA from control LNCaP and cells treated with *CYP1A1* siRNA#2 were evaluated for expression of apoptosis-related genes using the RT^2 Profiler™ PCR Array (Qiagen) by real-time PCR

Gene	Fold Difference
CD70	3.84
MCL1	2.07
TNFRSF11B	2.03
FAS	0.49
BCL2	0.37

Data presented are genes increased or decreased two-fold or greater due to *CYP1A1* knockdown in LNCaP cells.
